# Over-expression of Thioredoxin-1 mediates growth, survival, and chemoresistance and is a druggable target in diffuse large B-cell lymphoma

**DOI:** 10.18632/oncotarget.463

**Published:** 2012-03-23

**Authors:** Changping Li, Michael A. Thompson, Archito T. Tamayo, Zhuang Zuo, John Lee, Francisco Vega, Richard J. Ford, Lan V. Pham

**Affiliations:** ^1^ Department of Hematopathology, The University of Texas MD Anderson Cancer Center, Houston, TX; ^2^ Department of ProHealth Care Regional Cancer Center, Waukesha Memorial Hospital, Waukesha, WI

**Keywords:** Trx-1, chemoresistance, DLBCL, cellular redox

## Abstract

Diffuse Large B cell lymphomas (DLBCL) are the most prevalent of the non-Hodgkin lymphomas and are currently initially treated fairly successfully, but frequently relapse as refractory disease, resulting in poor salvage therapy options and short survival. The greatest challenge in improving survival of DLBCL patients is overcoming chemo-resistance, whose basis is poorly understood. Among the potential mediators of DLBCL chemo-resistance is the thioredxoin (Trx) family, primarily because Trx family members play critical roles in the regulation of cellular redox homeostasis, and recent studies have indicated that dysregulated redox homeostasis also plays a key role in chemoresistance. In this study, we showed that most of the DLBCL-derived cell lines and primary DLBCL cells express higher basal levels of Trx-1 than normal B cells and that Trx-1 expression level is associated with decreased patients survival. Our functional studies showed that inhibition of Trx-1 by small interfering RNA or a Trx-1 inhibitor (PX-12) inhibited DLBCL cell growth, clonogenicity, and also sensitized DLBCL cells to doxorubicin-induced cell growth inhibition in vitro. These results indicate that Trx-1 plays a key role in cell growth and survival, as well as chemoresistance, and is a potential target to overcome drug resistance in relapsed/refractory DLBCL.

## INTRODUCTION

Non-Hodgkin lymphoma (NHL) is the fifth most common cancer in the United States, and its incidence continues to increase. NHL affects all ethnic, racial, and gender groups approximately equally. Diffuse large B-cell lymphoma (DLBCL) is the most common type of NHL, with ~30,000 new cases per year in the US. DLBCL is initially chemoresponsive, with an overall response (complete response or partial response) rate of ~80% to frontline R-CHOP (rituximab, cyclophosphamide, doxorubicin, vincristine, and prednisone) chemo-immunotherapy. However, DLBCL frequently recurs (in ~40-50% of patients; usually within 2-3 years, depending on patient risk factors) as relapsed/refractory (r/r), incurable DLBCL with poor survival rates after current, inadequate salvage therapy regimens.[1]

“High-impact” studies in DLBCL should use novel therapeutic approaches to target the current large, heterogeneous, but poorly characterized population of patients with r/r DLBCL. The development of resistance to DLBCL therapy is currently the most challenging impediment to effective DLBCL therapy; the rapid development of multidrug resistance to structurally and functionally unrelated cancer drugs results in the early demise of nearly half of DLBCL patients.[2] Although progress in reversal of multidrug resistance, to result in effective therapy, has been slow and often disappointing in recent years,[3] new approaches to this daunting problem are essential for the ever-increasing numbers of patients with r/r DLBCL. The elucidation of molecular pathways and tumor-encoded genes whose expression contributes to the intrinsic resistance and the rapid growth of DLBCL cells could yield immediate clinical benefits and reveal new therapeutic targets for effective control and treatment of r/r DLBCL.

The thioredoxin (Trx) system is a major antioxidant system that is integral to maintaining the intracellular redox state. It contains thioredoxin-1 (Trx-1, TXN), a low-molecular-weight (10–12-kDa) cellular redox protein found in the nucleus and cytoplasm. Trx-1 regulates the activity of various enzymes, including those that function to counteract oxidative stress within the cell.[4] In addition, intracellular Trx-1 exerts most of its antioxidant properties by scavenging of reactive oxygen species. Moreover, intracellular Trx-1 acts as a co-factor for several enzymes and plays an important role in the regulation of redox-sensitive transcription factors.[5] Trx-1 is a proto-oncogene that stimulates tumor growth and inhibits both spontaneous and drug-induced apoptosis.[6] Increased Trx-1 gene expression is also associated with increases in both hypoxia-induced factor 1α (HIF-1α) levels and HIF-1 transactivation in cancer cells,[7] resulting in increased VEGF production and enhanced tumor angiogenesis.[8] In addition, overexpression of Trx-1 has been correlated with aggressive tumor growth, poorer prognosis, and decreased survival in patients with solid tumors.[9] Trx-1 appears to have an important role in maintaining the transformed phenotype of some human cancers as well as their resistance to chemotherapeutic drugs and is thus a rational target for cancer drug development. Trx was originally identified as an autocrine growth factor in transformed lymphoid cells.[10, 11] Increased Trx expression has been implicated in increased proliferation in various tumor types and model systems. However, there is a lack of supporting experimental evidence about the physiological and therapeutic significance of the Trx family in DLBCL. Recent preclinical in vitro data and clinical data in solid tumors strongly support the notion that the Trx system is of importance and that the development of drugs acting via the Trx system is a promising route, particularly for aggressive r/r DLBCL.

In this study, we showed that DLBCL tumors express higher basal levels of Trx-1 than normal B cells (by both western blotting and real-time PCR) and that Trx-1 expression level was significantly associated with decreased overall survival duration in DLBCL patients. Therefore, we hypothesize that Trx-1 plays an important role in the biology of DLBCL, particularly in regulating key growth/survival and chemoresistance mechanisms. The experimental design for this study was to characterize the expression of Trx-1, at both the mRNA and protein levels, in DLBCL and to thereby elucidate the functional significance of Trx-1 in the biology of DLBCL. Our results show that Trx-1 not only controls cell growth and survival but also regulates chemoresistance in the pathophysiology of DLBCL, and that targeting Trx-1 in DLBCL may have therapeutic significance.

## RESULTS

### Trx-1 is overexpressed in DLBCL

**Figure 1:**
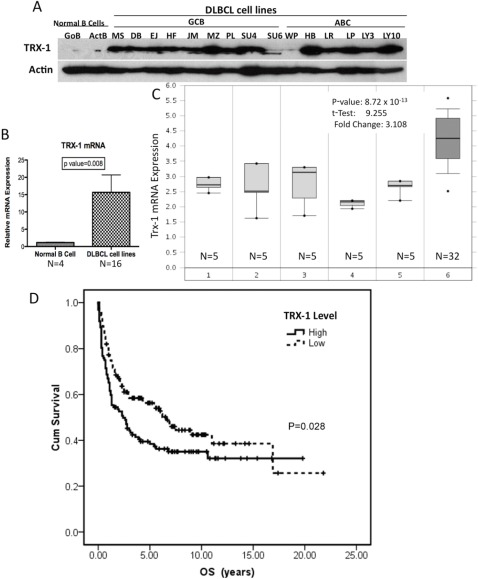
Trx-1 is highly expressed in DLBCL cell lines (A) Whole-cell extracts (50 μg) purified from normal B cells (quiescent [GoB] and activated [ActB]) and DLBCL cell lines (GCB subtype: MS, DB, EJ, HF, JM [McA], MZ, PL, SU4 [SUDHL-4], SU6 [SUDHL-6], and WP; ABC subtype: HB, LR, LP, LY3 [OCI-LY3], and LY10 [OCI-LY10]) were used to analyze for Trx-1 and actin (loading control) protein expression by western blotting. (B) Purified mRNA from normal B cells (n = 4) and DLBCL cell lines (n = 16) was subjected to RT-PCR. Statistical analysis was performed using the Student *t* test. P < 0.05 indicates significance. (C) Microarray data analyses of Trx-1 mRNA expression in primary DLBCL and normal B cells. Stages: 1, B-lymphocyte; 2, centroblast; 3, memory B-lymphocyte; 4, naïve pre-germinal center B-lymphocyte; 5, small cleaved follicular center cell; 6, DLBCL. The Student *t* test was conducted using the Oncomine software. The boxes represent the 25th through 75th percentiles, the horizontal lines represent the medians, the whiskers represent the 10th and 90th percentiles, and the asterisks represent the ends of the ranges. (D) Overall survival according to TXN expression level in Rosenwald's study cohort.^14^ Gene expression data and patient data were downloaded from http://llmpp.nih.gov/DLBCL/. Survival information was available for only 240 patients. These patients were divided into “high” (n = 112) and “low” (n = 128) groups according to whether their TXN levels were above or below the median expression level of the whole cohort. Overall survival of each group was estimated with a Kaplan–Meier plot, and the groups were compared using the log-rank test.

We first evaluated the expression of Trx-1 in DLBCL cells, both in cell lines and in primary tumor cells, compared with normal B lymphocytes. We found that Trx-1 protein is overexpressed in DLBCL cell lines, of both the activated B-cell-like (ABC) and germinal center B-cell-like (GCB) subtypes, compared with G0 naïve (unstimulated) or activated B cells (Figure 1A). Quantitative RT-PCR confirmed that Trx-1 mRNA expression is significantly higher (*P* = .008) in DLBCL cell lines than in normal B cells (Figure 1B). We then analyzed the expression levels of Trx-1 in primary DLBCL cells using Oncomine (https://www.oncomine.org), a publicly available cDNA cancer microarray database. Analysis of a representative data set (from Basso et al)[12] indicated that Trx-1 mRNA levels were higher in primary DLBCL cells than in normal B cells at different stages (Figure 1C). We also analyzed the Trx-1 gene expression profile in other types of lymphoid malignancies, and the results indicated that other types of lymphoma also have high expression of Trx-1 mRNA (Figure S1). Further analysis of other lymphoma profiling data sets (from Rosenwald et al[13, 14] and Alizadeh et al[15]) also showed high expression of Trx-1 in DLBCL, particularly the ABC subtype (Figure S2). We then examined the clinical significance of Trx-1 overexpression in primary DLBCL cells by using gene expression microarray analysis of a 240-sample data set with known clinical profiles.[13] Elevated Trx-1 expression was found to be significantly associated with decreased cumulative overall survival rate (*P* = .028; Figure 1D). These results suggest that Trx-1 is overexpressed in DLBCL, indicating that Trx-1 likely plays a key role in the pathobiology of DLBCL.

### TMA analysis of Trx-1 protein expression in DLBCL

**Figure 2:**
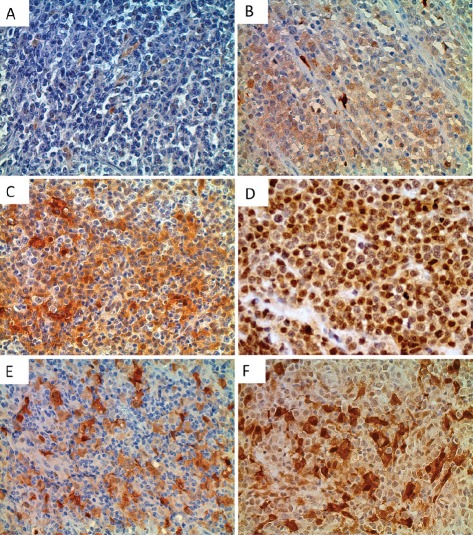
Immunohistochemical detection of Trx-1 protein levels in human DLBCL and normal lymphoid tissues Tissue sections from human DLBCL TMAs (TMA1 and TMA2, consisting of 92 and 47 cases, respectively) were examined immunohistochemically using an anti-Trx-1 antibody. Representative photomicrographs of negative (A), weak (B), intermediate (C), and strong (D) Trx-1 staining in DLBCL tumor cells. Original magnification, 400×. Representative photomicrographs of Trx-1 staining in stromal cells with Trx-1-negative tumor cells (E) or with Trx-1-positive tumor cells (F).

**Figure 3:**
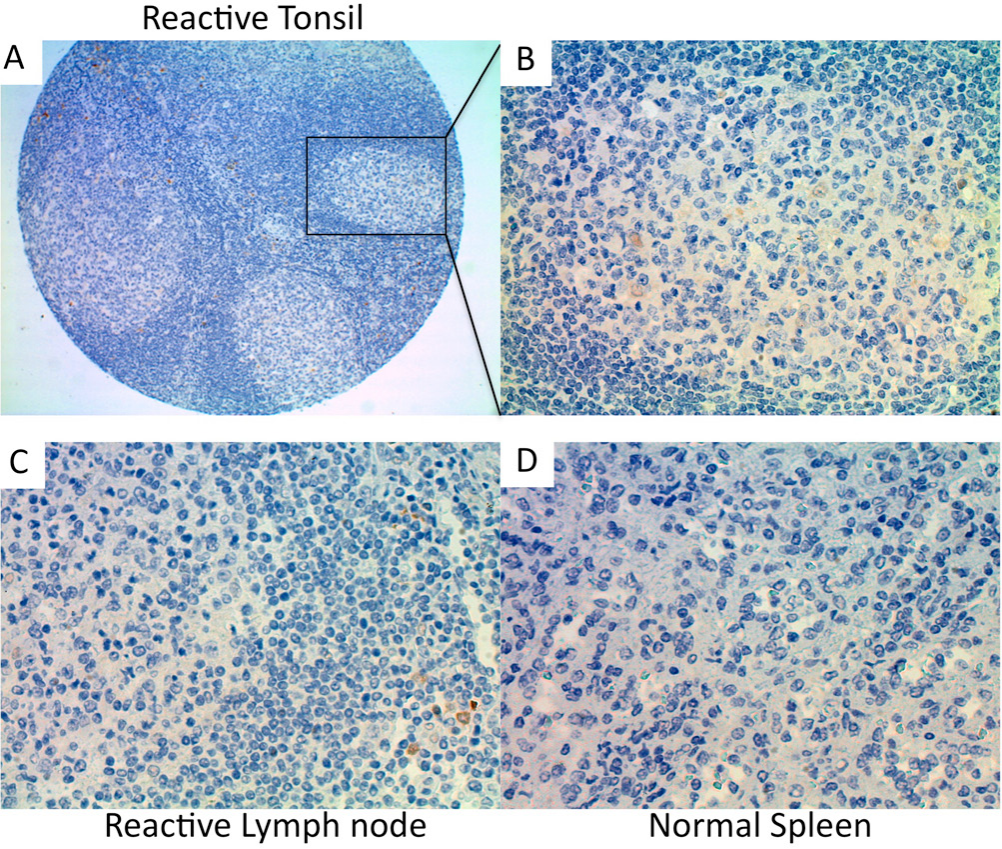
Immunohistochemical detection of Trx-1 protein levels in human normal lymphoid tissues (A) Reactive tonsil (10×), (B) reactive tonsil (400×), (C) reactive lymph node (400×), (D) normal spleen (400×).

Next, we performed immunohistochemical analysis of Trx-1 on TMAs of primary DLBCL and normal lymphoid tissues. Two TMAs comprising primary DLBCL were used: a commercially available TMA consisting of 92 cases but with no clinical data (TMA1, US Biomax) and a TMA produced at MD Anderson Cancer Center consisting of 47 cases with clinical data (TMA2). Figure 2A-F shows representative cases from these TMAs with negative and positive Trx-1 staining. Trx-1 was found to be highly expressed not only in tumor cells (Figure 2D) but also in histiocytes in the surrounding tumor microenvironment with macrophage- or fibroblast-/dendritic-like morphology (Figure 2E-F). The pattern of Trx-1 expression in tumor cells and histiocytes was, in most cases, in both the cytoplasm and the nucleus. When we combined results from both TMAs, the percentage of cases with negative, weak/intermediate, and strong Trx-1 staining in tumor cells was 27%, 39%, and 34%, respectively (Tables 1 and 2). The total percentage of cases with positive Trx-1 staining in tumor cells was 73%. We found Trx-1-positive histiocyte involvement in 29% of the cases with Trx-1-negative tumor cells, 52% of the cases with weak/intermediate Trx-1 staining in tumor cells, and 43% of the cases with strong Trx-1 staining in tumor cells (Tables I and II). Trx-1-positive histiocyte involvement was 42%. In TMA2, there was no significant association was found between Trx-1 positivity/negativity and overall survival duration (data not shown). This lack of association may be due to the low number of cases available. However, we did find an association between Trx-1 expression level and relapse status: patients with strong Trx-1 expression were more likely to have experienced relapse (50%) than those with negative Trx-1 expression (31%) (Table II). These relapsed cases were independent of Trx-1-positive histiocyte involvement (data not shown). We also evaluated Trx-1 expression in normal lymphoid tissues. In contrast to tumor cells, Trx-1 expression was not present in lymphocytes of reactive tonsil (Figure 3A-B), reactive lymph node (Figure 3C), or normal spleen (Figure 3D); however, some macrophage-like cells outside of the cortex as well as inside the follicles did stain positive for Trx-1 (Figure 3B). These results suggest that Trx-1 may play an intrinsic as well as an extrinsic (tumor microenvironment) role in the biology of DLBCL cells.

**Table I:** Immunohistochemical detection of Trx-1 protein levels in human DLBCL tissues

DLBCL TMA1	Negative	Weak/Intermediate	Strong	Total TRX-1 Positive
**Tumor cells**	22/92 (24%)	33/92 (36%)	37/92 (40%)	70/92 (76%)
**Histocytes involvement**	7/22 (32%)	18/33 (55%)	16/37 (43%)	41/92 (44%)

**Table II:** Immunohistochemical detection of Trx-1 protein levels in human DLBCL tissues

DLBCL TMA2	Negative	Weak/Intermediate	Strong	Total Positive
**Tumor cells**	16/47 (34%)	21/47 (45%)	10/47 (21%)	31/47 (66%)
**Histocytes Involvement**	4/16 (25%)	10/21 (48%)	4/10 (40%)	18/47 (38%)
**Cases Relapsed**	5/16 (31%)	9/21 (43%)	5/10 (50%)	19/47 (40%)

### Role of Trx-1 in cell growth and chemoresistance in DLBCL

**Figure 4:**
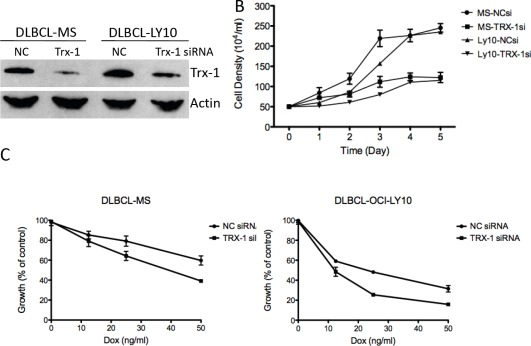
Inhibition of Trx-1 utilizing siRNA, inhibited DLBCL cell growth and sensitized DLBCL cells to Dox-induced growth inhibition in vitro (A) DLBCL cells (MS and OCI-LY10) were transfected with a negative control (NC) or Trx-1 siRNA. At 48 hours post-transfection, protein purified from transfected cells was subjected to western blotting for Trx-1 and actin. (B) DLBCL cells were transfected with a negative control (NC) siRNA or Trx-1 siRNA. Cells were counted using the trypan blue method at the indicated time point. (C) Cells from (B) were treated with increasing doses of Dox and were subjected to thymidine incorporation assays for 96 hours.

**Figure 5:**
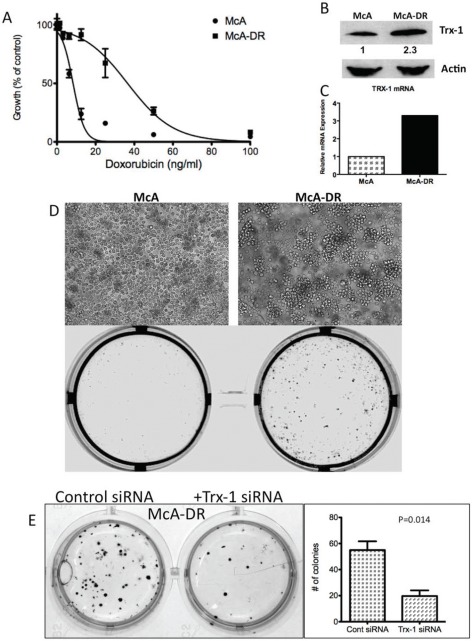
Trx-1 is overexpressed in a Dox-resistant cell line, McA-DR We generated a Dox-resistant human DLBCL cell line (McA-DR) from a parental cell line (McA) that was highly sensitive to Dox, by continuously exposing cells to gradually increasing doses of Dox. The resistant phenotype has been retained for more than 6 months despite growth in drug-free medium. (A) Proliferation assays of McA and McA-DR cells responding to Dox. (B) Trx-1 protein expression in McA and McA-DR cells by western blotting. Densitometry analysis of Trx-1 protein expression, showing that Trx-1 expression is ~2.3 times as high in McA-DR cells as in McA cells. (C) RT-PCR analysis showing overexpression of Trx-1 mRNA in McA-DR cells. (D) Light micrograph of McA and Dox-resistant clone (McA-DR) cell lines, and colony formation assays for McA and McA-DR cell lines. (E) McA-DR cells were transfected with a negative control or Trx-1 siRNA. Transfected cells were subjected to colony formation assays and incubated for 10–14 days. Colonies were photographed and counted.

To determine the biological significance of Trx-1 in the growth and survival of DLBCL, we used Trx-1 validated siRNA to knock down Trx-1 gene expression in two representative DLBCL cell lines, MS and OCI-LY10 (Figure 4A). siRNA against Trx-1 resulted in the inhibition of DLBCL cell growth by 50% after 3 days of incubation (Figure 4B). As our data showed that there is an association between the Trx-1 expression level and relapse status in DLBCL patients, we evaluated the chemoresistance mechanism of Trx-1 in DLBCL cell lines. First, we tested whether ablating Trx-1 expression in DLBCL cells can sensitize these cells to Dox, a main ingredient in frontline CHOP chemotherapy. Our data show that inhibition of Trx-1 expression sensitized DLBCL cells to Dox in two representative DLBCL cell lines (Figure 4C), suggesting that Trx-1 may play a role in drug resistance. Next, we generated a Dox-resistant (DR) DLBCL cell line (McA-DR) from a parental cell line (McA) that was initially highly sensitive to Dox (Figure 5A), by continuously exposing cells to gradually increasing doses of Dox. The resistant phenotype has been retained for more than 6 months despite growth in drug-free medium. We found that Trx-1 expression levels, both protein (Figure 5B) and mRNA (Figure 5C), were higher in the resistant cell line than in the parental cell line. In addition, we found that cells from the resistant cell line (McA-DR) formed cell clusters in culture medium and were more clonogenic in methylcellulose culture than the parental cell line (McA) (Figure 5D). Downregulation of Trx-1 by siRNA in the McA-DR cell line reversed the clonogenic activity (Figure 5E).

### Effect of the Trx-1 inhibitor PX-12 in DLBCL cells

**Figure 6:**
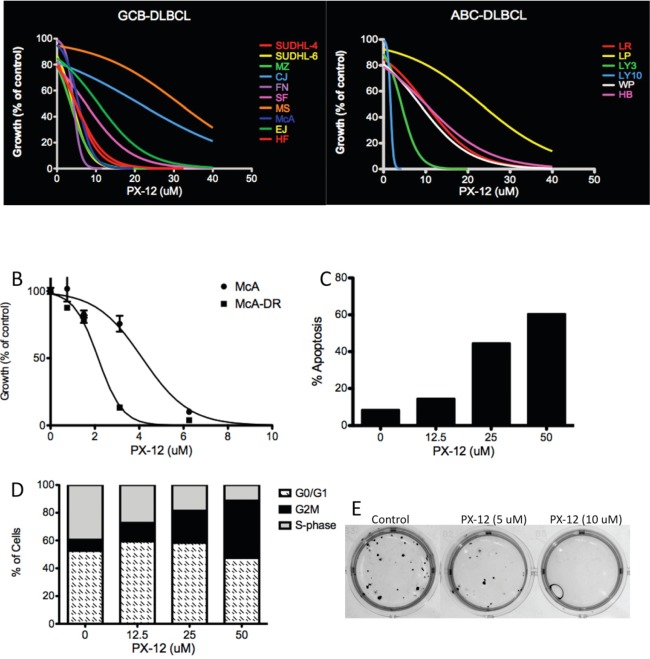
Effect of Trx-1 inhibitor PX-12 in DLBCL (A) Proliferation assays by thymidine incorporation in DLBCL cells treated with PX-12 (0–50 μM) for 96 hours. (B) Proliferation assays by thymidine incorporation in McA and McA-DR cells treated with PX-12 (0–10 μM) for 96 hours. (C) Apoptosis analysis by Annexin V/PI staining in McA-DR cells treated with PX-12. (D) Cells from (C) also underwent cell cycle analysis by PI staining and flow cytometry analysis. (E) Colony formation assays in methylcellulose in McA-DR control and PX-12-treated cells.

Additional experiments were performed to ascertain if pharmacologic agents targeting Trx-1 could recapitulate the effects of Trx-1 knock-down in DLBCL cells. PX-12 specifically inhibits Trx-1 by irreversibly thio-alkylating the Cys^73^ residue of Trx-1.[16, 17] PX-12 inhibition of Trx-1 has previously shown both excellent *in vitro antitumor activity* and promising *in vivo* antitumor activity in solid tumors.[18, 19] To test the efficacy of PX-12 in DLBCL cells, we exposed 18 DLBCL cell lines, including the Dox-resistant cell line McA-DR, to increasing concentrations (0–50 μM) of PX-12 and then analyzed cell proliferation using thymidine incorporation assays. The growth of all DLBCL cell lines was inhibited by PX-12 in a dose-dependent manner (Figure 6A), and the half maximal inhibitory concentration (IC_50_) value for PX-12 in each cell line was determined after testing a range of concentrations (0–50 μM) (Figure S3). Interestingly, the Dox-resistant cell line, McA-DR, was more sensitive to PX-12 than the parental cell line McA (Figure 6B). However, the mRNA level of Trx-1 does not correlate with PX-12 sensitivity in DLBCL cell lines (Figure S3B). In McA-DR cells, PX-12 induced apoptosis in a dose-dependent manner (Figure 6C) and induced cell cycle arrest at the G_2_M phase (Figure 6D). In McA-DR cells, treatment with the Trx-1 inhibitor PX-12 also inhibited the clonogenic activity (Figure 6E). These results suggest that PX-12 is a potential therapeutic agent that can reverse chemoresistance in DLBCL.

### Inhibition of Trx-1 activity modulates drug resistance gene expression in DLBCL cells

**Figure 7:**
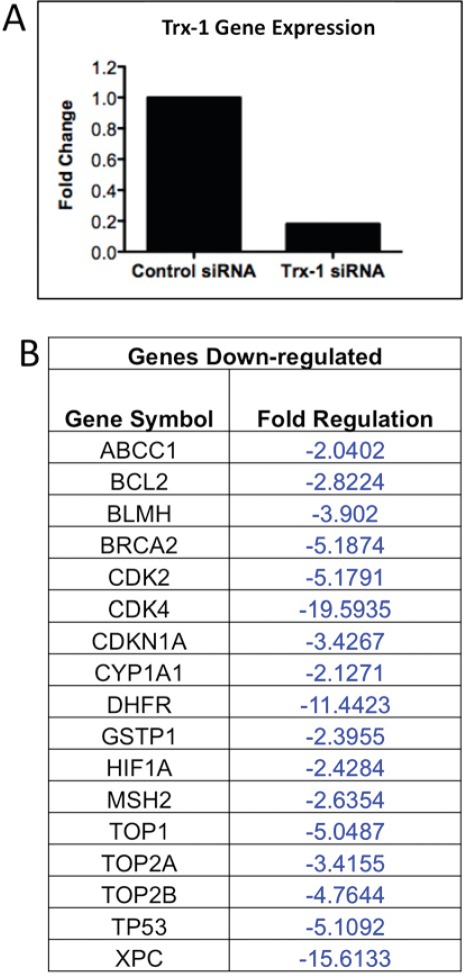
Inhibition of Trx-1 Activity modulates drug resistant genes expression in DLBCL cells (A) DLBCL-MS cells were transfected with a validated TXN siRNA. 48 hrs post-transfection, RNA was extracted and RT-PCR was performed to detect for TXN mRNA expression. (B) Purified RNA from control and TXN-siRNA-transfected cells were also used to perform RT-PCR array (Human Chemoresistance RT-PCR array from Sabioscienes). Genes with > 2-fold down-regulated in TXN knock-down cells compared to control cells are shown.

Because our data demonstrated that Trx-1 plays a role in chemoresistance of DLBCL, focused RT-PCR array techniques were used to examine if selective inhibition of Trx-1 modulated expression of genes involved in the body's response to chemotherapy in DLBCL cells. Of the 84 genes analyzed, 17 genes showed significant down-regulation (>2-fold) after Trx-1 knock-down by siRNA in a representative DLBCL cell line (MS) (Figure 7). These include drug resistance genes (*ABCC1 (MRP-1), BCL-2, TOP1, TOP2A, TOP2B, and TP53),* drug metabolism genes (*GSTpi, CYP1A1, BLMH and DHFR),* DNA repair genes *(MSH2, BRCA2, and XPC),* cell cycle genes *(CDK2, CDK4, and CDKN1A)* and the transcription factor *HIF1A.* These data suggest that chemoresistance effects of targeted inhibition of Trx-1 in DLBCL cells are mediated, at least in part, by down-regulation of several key genes that are known to play a role in drug resistance mechanisms.

## DISCUSSION

Chemoresistance is a major impediment to the treatment of patients with r/r DLBCL. Efforts to reverse chemoresistance of refractory DLBCL cells have been largely unsuccessful. On the basis of our data, we conclude that overexpression of Trx-1 in DLBCL cell lines and primary cells is associated with growth and survival, as well as chemoresistance, and that targeting Trx-1 with the novel Trx-1 inhibitor PX-12 may reverse chemoresistance. We postulate that the acquisition of high Trx-1 expression, either in tumor cells or in cells of the tumor microenvironment in DLBCL, may have occurred during chemotherapy, in a stressful microenvironment, to enable the cells to better tolerate oxidative stress. As a result, high basal expression of Trx-1 in tumor cells or in stromal cells of the surrounding microenvironment may lead to the up-regulation of critical drug resistance genes and the development of chemoresistance.

Although the basis for the high expression of Trx-1 in DLBCL is still unclear, a recent study demonstrated that VDUP-1 (vitamin D3 upregulated protein 1; also called TXNIP [thioredoxin-interacting protein-1] or TBP-2 [Trx-binding protein-2]), is downregulated in DLBCL.[20] Trx-1 activity can be modulated by interaction with VDUP-1, suggesting that VDUP-1 is a natural physiological inhibitor of Trx-1.[21] The Lymphoma/Leukemia Molecular Profiling Project used microarray technology to define a molecular profile for patients with DLBCL and developed a molecular outcome predictor score that accurately predicted patient survival.[20] The results suggested that DLBCL patients with the worst prognosis, according to the outcome predictor score, had decreased expression of catalase, glutathione peroxidase, manganese superoxide dismutase, and VDUP-1. Patients with the worst prognosis seemed to combine decreased expression of antioxidant defense enzymes with increased Trx system function (the redox signature score).[20] In fact, our analysis of a recent lymphoma profiling data set of 240 cases also indicated a significant association between high Trx-1 expression and decreased overall survival rate. These studies suggest a direct involvement of VDUP-1 and Trx-1 in the biology of DLBCL, but further studies are required to decipher their relationship with respect to their function in chemoresistance.

Here, we found that Dox-resistant cells express higher levels of Trx-1 gene and that downregulation of Trx-1 in these cells reversed the chemoresistant phenotype, suggesting that the high expression of Trx-1 in DLBCL may have been acquired during chemotherapy. Elevated Trx levels have also been implicated in the resistance of tumor cells to several commonly used chemotherapeutic agents, namely cisplatin, docetaxel, and anthracyclines (Dox).[22, 23] A gain of Trx-1 locus has been observed in drug-resistant cells identified by array CGH analysis,[24] suggesting that this locus is prone to genomic imbalances imposed by chemotherapy. High Trx-1 levels have also been shown to favor enhanced tumor cell survival and, in some studies, have been associated with poor prognosis in cancer patients.[25, 26] Although the mechanism of the chemoresistance function of Trx-1 is still unclear, Trx-1 itself regulates the activity of various transcription factors, including NF-κB, which is known be highly activated in DLBCL.[6] Our Trx-1 knock-down experiments showed that HIF-1α is a down-stream target gene of Trx-1, consistent with previous studies.[8] These results also show that Trx-1 can modulate several important genes that have shown to be involved in the pathophysiology of DLBCL or have had negative prognostic value in DLBCL patients, including bcl-2,[27] TOP2a,[28] GST-pi,[29] CYP1A1,[30] TP53,[31] and CDK's.[32-34] Of these genes, bcl-2,[35] GST-pi,[36] and TP53[37] are direct targets of Trx-1 in other systems. Clearly, coordinated down-regulation of several Trx-1 potential target genes, either directly or indirectly, could be responsible for protecting DLBCL cells against anticancer drugs. However, the contribution of individual Trx-1 target genes in chemoresistance may be different. Further investigation is warranted and necessary to determine the signaling components associated with Trx-1 and related downstream targets involved in growth/survival and chemoresistance in the pathophysiology of DLBCL.

Interestingly, we also found that Trx-1 is highly expressed not only in DLBCL tumor cells but also in stromal cells in the surrounding tumor microenvironment. This result is quite timely as a recent study demonstrated that stromal cells in the lymph node microenvironment of B-cell chronic lymphocytic leukemia (B-CLL) patients strongly expressed Trx-1 and produced soluble Trx-1 that can rescue CLL cells from apoptosis in vitro.[38] It has also been shown that Trx-1 is secreted by B lymphocytes,[10] monocytes,[39] regulatory T cells,[40] and a variety of cancer cells, and Trx-1 has previously been shown to exhibit cytokine-like properties by stimulating and activating normal B lymphocytes.[10] Soluble Trx-1 could function as an autocrine growth factor for human normal and at lease some malignant lymphoid cells.[41] Other studies have also suggested that soluble Trx-1 acts synergistically with CD40 stimulation to induce S-phase entry and mitosis in normal B cells and B-CLL.[41] The CD40 pathway has been implicated in the pathophysiology of the disease process of DLBCL[42-45] and has been shown to play a key role in chemoresistance.[46] These studies imply that the pre-existing activated CD40 pathway in concert with the extrinsic (tumor microenvironment signals) and intrinsic roles of Trx-1 in DLBCL cells could be important features that appear to provide cell growth and survival, as well as chemoresistance, advantages in DLBCL, particularly in r/r DLBCL. Therefore, the use of drugs, like PX-12, that inhibit the redox activity of Trx-1 might offer a novel approach to reverse chemoresistance in relapsed DLBCL. In fact, PX-12 has been used in human phase I and II clinical trials in solid tumors. PX-12 is well tolerated in solid tumors, and with our preclinical rationale we may now be able to explore the utility of this Trx-1 inhibitor alone or more likely in combination with newly identified cytotoxic or novel agents in reversing resistance and treating patients with r/r DLBCL.

This study has provided important new advances in understanding the biology of DLBCL chemoresistance, a very important but largely unknown, challenging area of lymphoma therapeutics, which may lead to the development of new, more effective approaches to treating this most common type of lymphoid cancer, particularly the increasing r/r form of DLBCL, which is currently incurable and rapidly leads to the patient's demise.

## MATERIAL AND METHODS

### Cells and reagents

Human DLBCL cell lines (MS, DS, DB, JM [McA], FN, EJ, HF, HB, MZ, LR, CJ, LP, and PL) were established from tissue biopsy or effusion specimens from patients as described elsewhere.[47] The SUDHL-4, SUDHL-6, OCI-LY3, and OCI-LY10 DLBCL cell lines were obtained from Dr. Michael Rosenblum (The University of Texas MD Anderson Cancer Center, Houston, TX). The BJAB cell line was obtained from American Type Culture Collection (ATCC) (Manassas, VA). This study was conducted in accordance with the Declaration of Helsinki and approved by the Institutional Review Board of MD Anderson Cancer Center. Informed consent was obtained from all patients whose tumor samples were used. The cells were cultured in RPMI-1640 medium (Gibco, Rockville, MD) containing 15% fetal calf serum (FCS) (HyClone Laboratories, Logan, UT). The monoclonal Trx-1 antibody and the Trx-1 inhibitor PX-12 (1-methylpropyl 2-imidazolyl disulfide) were provided by Dr. Garth Powis.[16, 19] The Trx-1 validated small interfering RNA (siRNA) and control siRNA were purchased from Applied Biosystems/Ambion (Austin, TX).

### TMA analysis and immunohistochemistry

We used two DLBCL tissue microarrays (TMAs). TMA1, consisting of 92 cases of DLBCL (TMA LY1001; a single core per case), was purchased from US Biomax (Rockville, MD), and TMA2, consisting of 47 cases of DLBCL, was provided by Dr. Francisco Vega.[48] The TMA slides were dewaxed at 55°C for 20 minutes, followed by three 5-minute washes with xylene. Immunostaining was performed using 5-μm-thick, formalin-fixed, paraffin-embedded tissue sections, epitope retrieval with Diva deblocking buffer and a deblocking chamber, and the Mach 3 system (all from Biocare Medical, Concord, CA). Staining was performed using the Autostainer Plus (DakoCytomation, Carpinteria, CA). The washing buffer used was 0.05 M Tris (tris(hydroxymethyl)aminomethane)-buffered saline supplemented with 0.05% Tween. 3,3′-diaminobenzidine tetrahydrochloride was used as the chromogen (Liquid DAB+ substrate chromogen system, DakoCytomation), and all tissue sections were counterstained with hematoxylin. The evaluation of Trx-1 staining was semiquantitatively scored by 3 scientists (L.V.P., R.J.F., F.V.) TMA positivity for Trx-1 was defined as immunostaining in >30% of the cells. Trx-1 protein expression was scored as negative, weak, or strong depending on the staining signal intensity. TMA photomicrographs were captured using an Olympus BX41 dual-head light microscope equipped with an Olympus Q-Color5 digital camera (Olympus America, Melville, NY), with a 20× plan apochromat objective. Digital images were obtained and adjusted using Adobe Photoshop CS3 (Adobe Systems).

### Proliferation assays

In vitro thymidine incorporation (proliferation) assays were performed as described previously.[49] Briefly, cells were plated (in triplicate) at 4.0 × 10^4^ cells/well in 200 μL of RPMI 1640 with 15% FCS and the indicated reagents in a 96-well plate and incubated in 5% CO_2_ at 37°C. After 48 hours, each well was pulsed with 0.5 μCi/10 μL of [^3^H]thymidine (Amersham, Arlington Heights, IL) for 16 hours. Cells were harvested, and the radioactivity was measured.

### Immunoblot analysis

Whole-cell extracts were solubilized with 1.0% SDS sample buffer and electrophoresed on a 4–15% SDS-PAGE gel (Bio-Rad, Richmond, CA). Proteins were transferred onto a polyvinylidene difluoride membrane and were probed with various specific primary antibodies and the appropriate horseradish peroxidase-conjugated secondary antibodies. Proteins were visualized using the ECL system (Amersham, Piscataway, NJ).

### RNA isolation and real-time PCR

Total RNA isolation was performed by using Trizol LS Reagent (Invitrogen) according to the manufacturer's instructions. Reverse transcription of RNA was carried out with a cDNA archive kit (Applied Biosystems, Foster City, CA). Synthesized cDNA was subjected to real-time polymerase chain reaction (RT-PCR) for the detection of related gene transcripts (Trx-1 and 18S). In brief, 2.5 μL of cDNA was placed in a 25-μL reaction volume containing 12.5 μL of TaqMan Universal PCR Master Mix, No AmpErase UNG, 8.75 μL of water, and 1.25 μL of primers and probe sets. The primers and probes were purchased from Applied Biosystems and were designed to span exon–exon boundaries. The Human Cancer Drug Resistance and Metabolism PCR Arrays were purchased from SAbiosciences (Frederick, MD). Amplification was performed in the ABI Fast 7500 Real-Time PCR system (Applied Biosystems) using the following cycling program: 95°C for 10 minutes; 40 cycles of 95°C for 15 seconds, 60°C for 60 seconds. All samples were analyzed in triplicate. DNA contamination was evaluated by performing PCR on the non-reverse-transcribed control of each sample. The relative expression levels of the genes of interest were normalized to the endogenous reference 18S and relative to a control sample as a calibrator by using the formula 2^−ΔΔCT^. The threshold cycle (CT) reflects the cycle number at which the fluorescence generated within a reaction crosses the threshold.

### Establishment of doxorubicin-resistant cell lines

We generated a doxorubicin-resistant (DR) DLBCL cell line (McA-DR) from a parental cell line (McA) by multistep exposures of cells to increasing doses (up to 50 ng/mL) of doxorubicin (Dox) for 8 weeks. Briefly, cells were initially cultured in a low drug concentration for 1 week and then maintained in drug-free medium for 1 week to stabilize the cells. Medium with increasing drug concentration was changed every other week during the selection, and subsequently the cells became resistant to Dox. The resistant clones were expanded in drug-free media. Expanded clones were retested for drug resistance before any further studies.

### Transient transfection of DLBCL cells

DLBCL cells (MS and OCI-LY10) were transiently transfected with 50 nM Trx-1 validated siRNA (Applied Biosystems/Ambion) using the Neon transfection system (Invitrogen, Carlsbad, CA).[50] The transfection efficiency ranges from 70% to 80% with 75% cell viability for MS cells, and from 50% to 60% with 70% viability for OCI-LY10 cells.

### Methylcellulose clonogenic assays

The colony formation assay in methylcellulose (M3434; StemCell Technologies, Vancouver, BC, Canada) was performed according to the manufacturer's instructions. Briefly, DLBCL cells were initially plated in methylcellulose at a density of 2 × 10^3^ cells/0.3 mL/well in a 12-well plate and incubated for 10–14 days. Colonies were stained with p-iodonitrotetrazolium violet, photographed, and counted using QCapture Pro software (QImaging, Surrey, BC, Canada). All experiments were performed in duplicate and repeated at least 3 times.

Apoptosis analysis by Annexin V/propidium iodide (PI) staining and cell cycle analysis by PI staining and flow cytometry analysis methods were previously described.[49]

### Statistical Analysis

The software used for statistical analysis was GraphPad Prism 5b (GraphPad Software, Inc., La Jolla, CA). Statistical significance was determined by the student *t*-test. P-values <0.05 were considered statistically significant.
